# Antioxidant and Proapoptotic Activities of *Sclerocarya birrea* [(A. Rich.) Hochst.] Methanolic Root Extract on the Hepatocellular Carcinoma Cell Line HepG2

**DOI:** 10.1155/2015/561589

**Published:** 2015-05-17

**Authors:** Maria Francesca Armentano, Faustino Bisaccia, Rocchina Miglionico, Daniela Russo, Nicoletta Nolfi, Monica Carmosino, Paula B. Andrade, Patrícia Valentão, Moussoukhoye Sissokho Diop, Luigi Milella

**Affiliations:** ^1^Department of Science, Basilicata University, Viale dell'Ateneo Lucano 10, 85100 Potenza, Italy; ^2^REQUIMTE/LAQV, Laboratório de Farmacognosia, Departamento de Química, Faculdade de Farmácia, Universidade do Porto, Rua de Jorge Viterbo Ferreira No. 228, 4050-313 Porto, Portugal; ^3^Faculte des Sciences et Techniques, Universite Cheikh Anta Diop de Dakar, BP 5005, Dakar-Fann, Senegal

## Abstract

The main goal of this study was to characterize the *in vitro* antioxidant activity and the apoptotic potential of *S. birrea* methanolic root extract (MRE). Among four tested extracts, obtained with different solvents, MRE showed the highest content of polyphenols, flavonoids, and tannins together with antioxidant activities tested with superoxide, nitric oxide, ABTS, and beta-carotene bleaching assays. Moreover, the cytotoxic effect of MRE was evaluated on the hepatocarcinoma cell line HepG2. In these cells, MRE treatment induced apoptosis and generated reactive oxygen species (ROS) in dose-dependent manner. The cytotoxic effect promoted by MRE was prevented by pretreatment of HepG2 cells with N-acetyl-L-cysteine (NAC), suggesting that oxidative stress was pivotal in MRE-mediated cell death. Moreover, we showed that the MRE treatment induced the mitochondrial membrane depolarization and the cytochrome *c* release from mitochondria into the cytosol. It suggests that the apoptosis occurred in a mitochondrial-dependent pathway. Interestingly, MRE showed a sensibly lower cytotoxicity, associated with a low increase of ROS, in normal human dermal fibroblasts compared to HepG2 cells. It is suggested that the methanolic root extract of *S. Birrea* is able to selectively increase intracellular ROS levels in cancer cells, promoting cell death.

## 1. Introduction

Natural products have found many applications in the fields of medicine, pharmacy, and biology. A considerable number (approximately 60%) of currently used antitumor agents are molecules identified and isolated from plants or their synthetic or semisynthetic derivatives [1, 2]. Some natural compounds are able to trigger the apoptosis signalling system in cancer cells disturbing their proliferation [3, 4], though their molecular mechanisms of action are not always well understood.

It is well established that carcinogenesis is closely associated with elevated levels of intracellular free radicals (ROS/RNS) to drive proliferation and other events required for tumor progression. This event establishes a state of increased basal oxidative stress, making cells vulnerable to chemotherapeutic agents, including plant-derived polyphenols, that further increase ROS generation or that weaken cell antioxidant defenses [5].

*Sclerocarya birrea* (A. Rich.) Hochst., known as marula, is a savannah tree belonging to the Anacardiaceae family [6]. The marula tree has been the subject of numerous chemical, biological, and environmental investigations since 1906 [7]. It has been identified as one of five fruit tree species that should be integrated in the domestication process because it is an important food and medicinal source for rural areas [8, 9]. Different parts of the plant are traditionally used: the fruits are eaten or processed to make beer or jam; the kernels are eaten or used for oil extraction; the leaves are used as forage for livestock; the stem-bark, root, and leaf extracts of* S. birrea* are used against human ailments [6]. Hamza et al. (2006) have reported that methanolic extracts from* S. birrea* roots inhibited the growth of* Candida* spp. and* Cryptococcus neoformans* [9]. It was also demonstrated that methanol and water root extracts act as potent antioxidants [10, 11]. Moreover, water and acetone extracts of* S. birrea* stem bark showed anticancer and proapoptotic activities [12].

The aim of this study was to examine the efficacy of* S. birrea* methanolic root extract (MRE) as an antioxidant, using* in vitro* assays. Additionally, its cytotoxic activity on the human hepatocarcinoma cell line HepG2 was evaluated here for the first time.

Obtained results show that MRE presents a strong antioxidant activity* in vitro* and a prooxidant activity in cells. Moreover, this extract shows higher ROS-mediated cytotoxic effect in HepG2 cells compared to normal human fibroblasts, suggesting its possible use for selectively killing malignant cells [5].

## 2. Materials and Methods

### 2.1. Plant Materials

Roots of* Sclerocarya birrea* (Anacardiaceae family) were collected in Senegal in September 2010. Roots were cleaned to remove foreign particles, cut into small pieces, and dried at room temperature. Plant material was successively extracted with 5 volumes (v/w) of* n*-hexane, chloroform, chloroform : methanol 9 : 1, and methanol by a sequential maceration, as described previously [11], obtaining 4 extracts with increasing polarity: *n*-hexane (HRE), CHCl_3_ (CRE), CHCl_3_ : MeOH (9 : 1) (CMRE), and MeOH (MRE) root extracts. Extracts were dried and stored at 4°C until the use.

### 2.2. Chemicals

Folin Ciocalteu's reagent, sodium carbonate (Na_2_CO_3_), aluminium chloride (AlCl_3_), sodium nitrate (NaNO_3_), sodium hydroxide (NaOH), bovine serum albumin (BSA), sodium dodecyl sulphate (SDS), triethanolamine, iron(III) chloride (FeCl_3_), ABTS [2,2′-azinobis(3-ethylbenzothiazoline-6-sulfonic acid)], potassium persulfate, *β*-carotene, linoleic acid, Tween 20, ascorbic acid, sodium nitroprusside (SNP), sulfanilamide, naphthylethylenediamine, nicotinamide adenine dinucleotide (NADH), phenazine methosulphate (PMS), nitroblue tetrazolium (NBT), potassium phosphate monobasic (KH_2_PO_4_), gallic acid, quercetin, tannic acid, 6-hydroxy-2,5,7,8-tetramethylchromane-2-carboxylic acid (trolox), butylated hydroxytoluene (BHT), Dulbecco's Modified Eagle Medium (DMEM), dimethyl sulfoxide (DMSO), Calcein-AM, 2′,7′-dichlorodihydrofluorescein diacetate (DCFH-DA), Hoechst 33258 solution, and N-acetyl-L-cysteine (NAC) were purchased from Sigma-Aldrich (Milan, Italy). Solvents as acetic acid (CH_3_COOH), hydrochloric acid (HCl), chloroform (CHCl_3_), and phosphoric acid (H_3_PO_4_) were purchased from Carlo Erba Reagents (Milan, Italy).

Trypsin-EDTA solution, FBS, glutamine, penicillin-streptomycin, and PBS were purchased from Euroclone (Milan, Italy). TMRM (Life Technologies) was a kind gift from Dr. M. Lasorsa (IBBE, CNR, Bari).

### 2.3. Total Phenolic Content

The total phenolic content (TPC) was determined for each extract, by Folin-Ciocalteu assay, as previously reported with slight modification [13]. Briefly, 75 *μ*L of diluted extract and 425 *μ*L of distilled water were added to 500 *μ*L Folin-Ciocalteu reagent and 500 *μ*L of Na_2_CO_3_ (10% w/v). The mixture was mixed and incubated for 1 h in the dark at room temperature. After incubation, the absorbance was measured at 723 nm. Gallic acid was used as standard to plot the regression curve. TPC was than expressed as mg gallic acid equivalent (GAE)/g of dried extract.

For all spectrophotometric measurements, a CARY 1E UV-VIS spectrophotometer (Varian, Leini, Italy) was used.

### 2.4. Total Flavonoid Content

An aliquot (150 *μ*L) of each extract was added to 45 *μ*L of 5% NaNO_3_ into microcentrifuge tube. In the fifth and in the sixth minute, respectively, 90 *μ*L of 10% AlCl_3_ and 300 *μ*L of 1 M NaOH solution were added. The final volume of the mixture was then brought to 1.5 mL by adding distilled water. The absorbance was measured against blank reagent at 510 nm after 10 minutes of incubation at room temperature [14]. Quercetin was used as standard to plot the regression curve. The total flavonoid content (TFC) was expressed as mg of quercetin equivalent/g of dried extract (mg QE/g of extract).

### 2.5. Total Tannin Content

To 250 *μ*L of each extract, 500 *μ*L of bovine serum albumin solution in 0.2 M acetic buffer, pH 5.0 with 0.17 M NaCl was added and mixed carefully [15]. After 15 min, samples were centrifuged at 5000 g for 15 min. The supernatant was removed, and the pellet was dissolved in 1 mL of aqueous solution containing 1% SDS and 4% triethanolamine. Then 250 *μ*L of 0.01 M FeCl_3_ in 0.01 M HCl was added. After 30 min, the absorbance was recorded at 510 nm. Total tannin content (TTC) was expressed as mg of tannic acid equivalent/g of dried extract (mg TAE/g of extract), in this case tannic acid was used to construct a regression curve.

### 2.6. ABTS Assay

The free radical scavenging capacity of each plant extract was also studied using the 2,2′-azinobis(3-ethylbenzothiazoline-6-sulfonic acid) diammonium salt (ABTS^•^) radical assay [16]. ABTS was dissolved in deionized water to a 7 mM concentration and its radical cation (ABTS^+•^) was produced by reacting ABTS solution with 2.45 mM potassium persulfate and allowing the mixture to stand in the dark at room temperature for 12–16 h before use. Each extract (75 *μ*L) was added to 1425 *μ*L of ABTS^+•^ solution and the absorbance was measured after 2 h of incubation in the dark. All solutions were fresh prepared for the analysis. Results are expressed as percentage of radical inhibition. Trolox was used as reference standard.

### 2.7. Beta-Carotene Bleaching Assay

The antioxidant activity was evaluated by the *β*-carotene-linoleic acid bleaching method (BCB) as previously described [17, 18]. The absorbance was measured at 470 nm. Results are expressed as percentage of antioxidant activity (% AA).

### 2.8. Nitric Oxide (NO^•^) Radical Scavenging Activity

The antiradical activity was determined spectrophotometrically, according to a previously described procedure [19]. EC50 was calculated from three independent assays, performed in triplicate. Results are expressed as percentage of radical inhibition. Ascorbic acid was used as positive control.

### 2.9. Superoxide Anion (**O**_**2**_^•−^) Scavenging Activity

The effect of each extract on the superoxide radical-induced reduction of NBT was monitored at 560 nm. Superoxide radicals were generated by the NADH/PMS system, as previously reported [19]. For each extract, different concentrations were tested. Results are expressed as percentage of radical inhibition. Ascorbic acid was used as positive control.

### 2.10. Cell Culture and Drug Treatment

The human hepatocellular carcinoma cell line HepG2 was kindly gifted from Dr. V. Infantino (University of Basilicata-Italy). HepG2 cells were cultured in DMEM (supplemented with 10% fetal bovine serum, 2 mM glutamine, 100 U/mL penicillin, and 100 *μ*g/mL streptomycin) and maintained at 37°C in a humidified atmosphere containing 5% CO_2_.

Normal human dermal fibroblasts (adult, HDFa, Life Technologies) were cultured in DMEM (supplemented with 10% fetal bovine serum, 2 mM glutamine, 100 U/mL penicillin, 100 *μ*g/mL streptomycin, and 1% nonessential amino acids) and maintained at 37°C in a humidified atmosphere containing 5% CO_2_. Cultures were routinely passed at 70–80% of confluence and, for this study, cultures were not expanded for more than 4–8 passages.

The methanolic root extract was dissolved in DMSO at 50 mg/mL as a stock solution and diluted to the required concentrations with fresh medium immediately before use. The final DMSO concentration in the cultures was 0.4% (v/v), which did not affect cell growth when compared with the vehicle-free controls. DMSO treated cells were used as control in all the experiments.

### 2.11. Cytotoxicity Assay

The cytotoxicity of* S. birrea* methanolic root extract was tested against HepG2 and normal human dermal fibroblasts cell lines using Calcein-AM viability assay. Calcein-AM is a nonfluorescent, hydrophobic compound that easily permeates intact, live cells. Once inside the cells, the hydrolysis of Calcein-AM by endogenous esterases produces calcein, a hydrophilic, highly negatively charged fluorescent compound that is well-retained in the cytoplasm of live cells. The fluorescent signal generated from the assay is proportional to the number of living cells in the sample. In brief, HepG2 and fibroblasts cells were seeded at a density of 1 × 10^4^/well in 96-well black-walled plates for 24 h and then treated with different concentrations of MRE (10, 50, 100, and 200 *μ*g/mL) for 24 h and 48 h. Moreover, HepG2 cells were pretreated with 10 mM NAC, added 1 h before each treatment. After discarding the medium from wells, 100 *μ*L of 1 *μ*M Calcein-AM in PBS was added to each well, incubating at 37°C for 30 min. The fluorescence was measured by GLOMAX Multidetection System (Promega, Madison, WI, USA) using blue filter (Ex 490 nm, Em 510–570 nm).

### 2.12. Observation of Morphological Changes

HepG2 cells were cultured as above, seeded in 12-well plates at a density of 2 × 10^5^ cells per well, and treated with extract at different concentrations (10, 50, 100, and 200 *μ*g/mL) for 24 h, with or without 10 mM of N-acetyl-L-cysteine (NAC), added 1 h before each treatment. The cellular morphology was observed using inverted phase contrast microscopy (Nikon Eclipse TS100).

Apoptosis was determined by the assessment of nuclear morphology using Hoechst 33258 DNA staining. Briefly, cells were seeded at a density of 2 × 10^5^/well in 12-well plates and were allowed to adhere overnight to glass coverslips. After treatment with root extract at different concentrations (10, 50, 100, and 200 *μ*g/mL) for 24 h, cells were fixed with 4% paraformaldehyde for 20 min, washed with PBS, and stained with 10 *μ*g/mL Hoechst 33258 at room temperature for 10 min in the dark. The cells were washed with PBS for morphologic observation by fluorescence microscopy (NIKON Eclipse 80i).

### 2.13. Measurement of Reactive Oxygen Species Generation

The intracellular level of ROS was determined using a cell-permeable fluorogenic probe, 2′,7′-dichlorofluorescein diacetate (DCFH-DA). This molecule is deacetylated by intracellular esterases and converted to nonfluorescent dichlorodihydrofluorescein (DCFH), which is oxidized rapidly to the highly fluorescent compound dichlorofluorescein (DCF) in the presence of ROS. HepG2 and fibroblasts cells were seeded into dark 96-well tissue culture plates at a density of 5 × 10^4^ cells/well treated with methanolic root extract (10, 50, 100, and 200 *μ*g/mL) for 3 h. Then, cells were stained with 10 *μ*M DCFH-DA for 30 min at 37°C in the dark and washed three times with PBS. The fluorescence was measured by GLOMAX Multidetection System (Promega, Madison, WI, USA) using blue filter (Ex 490 nm, Em 510–570 nm). For HepG2 cells, the experiment was performed also with 10 mM NAC, added 1 h before each treatment.

### 2.14. Annexin V/7-AAD Staining Assay

The percentage of cells undergoing apoptosis and necrosis after treatment with different concentrations of methanolic root extract was quantified using FITC Annexin V-7-AAD kit (BD Pharmingen). HepG2 cells were seeded at a density of 2 × 10^5^ cells/well in 12-well plates and treated with different concentrations of extract (50, 100, and 200 *μ*g/mL) for 24 h. The cells were harvested and resuspended in binding buffer and finally 5 *μ*L of Annexin V-FITC and 5 *μ*L of 7-AAD were added. Each tube was incubated in the dark for 15 min at room temperature. The stained cells were analysed on a FACS Canto II flow cytometer.

### 2.15. Measurement of Mitochondrial Membrane Potential (ΔΨ_*m*_)

The level of ΔΨ_*m*_ was monitored by flow cytometry (FACSCanto II) equipped with DIVA software (BD Biosciences, San Jose, CA) using the TMRM probe, a cell-permeant, cationic, red-orange fluorescent dye that is capable of selectively entering active mitochondria. Briefly, 2 × 10^5^ cells/well in 12-well plates were treated with different concentrations of root extract (10, 50, 100, and 200 *μ*g/mL) for 3 h. Cells were trypsinized, washed in ice-cold PBS, and incubated with 150 nM TMRM at 37°C for 20 minutes in darkness. Subsequently, cells were collected and diluted with PBS and then analysed by flow cytometry. Excitation wavelength was set at 488 nm and emission wavelength was collected at 575 nm.

### 2.16. Mitochondria Enrichment and Western Blotting

HepG2 cells were seeded into 100 mm dishes (1 × 10^7^ cells/dish) and then treated with 109 *μ*g/mL of root extract (IC_50_ value at 24 h) for different time periods (3 h, 6 h, and 24 h). Cells were harvested, resuspended in ice-cold isotonic buffer (0.25 M sucrose, 5 mM Tris-HCl, pH 7.5, and 1 mM EDTA), and homogenized using a glass Teflon homogenizer (35–40 times up and down). Unbroken cells and nuclei were sedimented by centrifugation at 600 ×g for 10 min. Supernatants were centrifuged at 10,000 ×g for 30 min, the supernatants (cytosolic fraction) were removed, and the mitochondrial pellets were resuspended in RIPA buffer (PBS pH 7.4, 1% NP-40, 0.5% sodium deoxycholate, and 0.1% sodium dodecyl sulfate) and supplemented with proteases and phosphatases inhibitor cocktail (Sigma). Protein concentration was measured using Bio-Rad Protein Assay (Bio-Rad, Hercules, CA, USA). Equal amounts of protein lysates were resolved on 4–17% SDS-PAGE and then blotted onto a nitrocellulose membrane (GE). The membrane was blocked with 5% nonfat dry milk in TBST buffer (100 mM Tris-HCl pH 7.5, 150 mM NaCl, and 0.05% Tween 20) for 1 h and then incubated overnight at 4°C with primary antibody against cytochrome* c* (1 : 2000, Abcam). After that, the membrane was washed three times with TBST buffer and incubated at room temperature for 1 h with anti-mouse horseradish-peroxidase-conjugated secondary antibody (1 : 3000, Sigma). Detection was performed using the enhanced chemiluminescence (ECL) kit (GE).

### 2.17. Statistical Analysis and Spectrophotometric Measurement

All the results are presented as mean ± SD of three independent experiments. In the viability assay, the percentage survival values were normalized by an arcsine square root transformation and then compared with analysis of variance (ANOVA) and Tukey's HSD test. In the measurement of reactive oxygen species and mitochondrial membrane potential, statistical significances were analysed by one-way analysis of variance (ANOVA) and Tukey's HSD test. Both analyses were performed using software R version 2.8.1 (R Development Core Team, 2008). Significant differences (*P* < 0.05) are denoted by different letters. In the assessment of apoptosis, we performed a chi-square test (*P* < 0.05 was considered significant). All statistical procedures related to antioxidant tests were computed using the statistical package Statistica for Windows (ver. 5.1., 1997) (Statsoft Inc., Tulsa, USA). To avoid the error due to extract absorbance, from each experimental measure, the absorbance of extract solubilized at the same concentration in the same solvent at the same wavelength was subtracted.

## 3. Results and Discussion

Numerous studies using cancer cell lines and animal models of carcinogenesis showed that among polyphenols, generally recognized as antioxidants, a wide range possesses anticancer and apoptosis-inducing properties [19–23]. In fact, it is well known that plant-derived antioxidant polyphenols possess dual prooxidative and antioxidative activities, depending on some factors such as their metal-reducing potential, chelating behavior, and pH ad solubility characteristics [24, 25].

Prominent is the goal to clarify the molecular mechanism whereby a plant-derived extract, rich in phenolic compounds, exerts an anticarcinogenic effect, through intrinsic and newly generated ROS, both of which are able to modulate chemical signaling pathways leading to apoptotic effects [26].

### 3.1. Total Phenols, Flavonoids, and Tannins Content of Extracts

*Sclerocarya birrea* is traditionally used for the treatment of various complaints and, as described above, several studies reported the relevant biological activities of different parts of this plant. A recent work underlined as* Sclerocarya birrea* extracts, with particular regard to the seed cake and root extracts, could be used as prophylactic antioxidant agents [9]. To select the most promising extract to be effective as antioxidant in* in vitro* assays, all root extracts were evaluated for their TPC, TFC, and TTC. Quantitative results demonstrated that crude methanol root extract (MRE) possesses the highest content of TPC, TFC, and TTC in comparison with other extracts (Table 1). MRE showed a content up to 20 times higher than HRE and sensibly higher than the others, with 861.94 ± 12.25 mg/g of total phenolics (GAE/g of extract), 95.47 ± 8.27 mg/g of total flavonoids (QE/g of extract) and 1109.68 ± 21.59 mg/g of total tannins (TAE/g of extract). Unlike previously reported [10], TPC measured in our study was sensibly higher. This is reasonably due to our extraction procedure: *n*-hexane, CHCl_3_, CHCl_3_ : MeOH (9 : 1), and MeOH versus *n*-hexane and 60% methanol described by Mariod et al. [10]. It is evident that our extraction procedure allowed the increase of phenolics in MRE.

### 3.2. Antioxidant Assays on Selected Extract

A preliminary screening of all extracts with antioxidant tests (data not shown) was performed and our results confirmed the effectiveness of MRE among others. In details, extract antioxidant activity, at different concentrations, was assayed by 4 different tests. It was previously underlined that at least two* in vitro* procedures should be carried out for the evaluation of extract antioxidant activities [27, 28]. In particular, MRE was evaluated firstly for its antiradical activity with the most popular ABTS method. ABTS decolorization assay is applicable for both hydrophilic and lipophilic antioxidants: the preformed radical monocation of ABTS^+•^ is generated by oxidation of ABTS with potassium persulfate and is reduced in the presence of such hydrogen-donating antioxidants. The EC50 calculated was 12.54 ± 0.47 *μ*g/mL.

**Figure 1:**
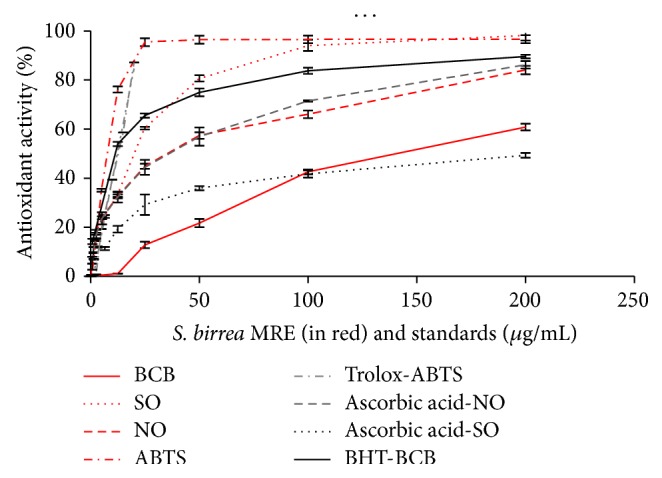
Antioxidant activity of* Sclerocarya birrea* methanolic root extract (MRE) compared with the reference standards. Antioxidant activity was measured by 4 different tests and in each one it is demonstrated to be dose-dependent. ABTS, nitric oxide (NO), superoxide anion (SO), and *β*-carotene bleaching (BCB) assays.

The antioxidant effect of the extract on the peroxidation of linoleic acid in the *β*-carotene/linoleic acid system was also investigated. The oxidation of linoleic acid generates Peroxyl free radicals, which will then oxidize the highly unsaturated *β*-carotene. The presence of antioxidants minimizes the oxidation of *β*-carotene. The ability of MRE to inhibit *β*-carotene bleaching was 60.29 ± 1.15% at 200 *μ*g/mL (Figure 1) while the EC50 was found to be 151.02 ± 4.72 *μ*g/mL.

Superoxide anion is a ROS normally produced inside the body. Controlled production of this radical is essential to maintain a healthy environment, but it is known to be very harmful to cellular components as a precursor of a more reactive oxygen species, for example, the hydroxyl radical [29]. The extract is found to be an efficient scavenger of superoxide radical generated in NADH/PMS system* in vitro* and its activity is comparable to that of ascorbic acid. The scavenging effect of the root extract was of 97.47 ± 2.52% at 200 *μ*g/mL concentration (Figure 1) and in this case the EC50 was 21.21 ± 2.14 *μ*g/mL.

MRE also caused a dose-dependent inhibition of nitric oxide (Figure 1): it is evident a nitric oxide scavenger activity (78.08 ± 3.24% at 200 *μ*g/mL). It is well known that nitric oxide is involved in many physiological processes and it is also implicated in inflammation, cancer, and other pathological conditions [30, 31]. NO has both cytoprotective and cytotoxic role. Its cytotoxic activity is related to the production of peroxynitrite ions when it reacts with O_2_^−^ ions. These compounds are responsible for altering the structural and functional behavior of many cellular components. In aqueous solution, at the physiological pH, SNP spontaneously generates nitric oxide that interacts with oxygen to produce nitrite, which can be determined by Griess reaction. EC50 calculated for NO assay was 32.18 ± 3.24 *μ*g/mL. Radical scavenging tests showed that MRE has an interesting antioxidant and dose-dependent activity (Figure 1). Considering the EC50 values of all radical scavenging tests, we can assess that MRE has a noticeable effect on cationic and anionic radicals. The lower lipid peroxidation activity is probably due to the characteristics of this assay that resulted in being more suitable for more lipophilic compounds [18].

None of the antioxidant assays (NO, SO, or ABTS test) was previously reported for measuring the activity of* S. birrea* methanolic root extract. The phytochemical investigation, reported by Russo et al. [11], demonstrated that roots of* S. birrea* are principally constituted by galloylated tannins and this is congruent with our results. In fact, the presence of galloyl groups at the 3 position plays an important role in antioxidant and protective activity [32] and it justifies the high antioxidant activity of MRE.

These results agree with those available in literature in which a direct correlation between phenolic compound levels in the extracts and their* in vitro* antioxidant activities was found [10, 18, 33].

### 3.3. Cytotoxic Effect of Methanolic Root Extract on HepG2 Cells

The neoplastic evolution needs both a deregulation of cell proliferation and a suppression of apoptosis, so both cellular processes represent obvious targets for therapeutic intervention in all cancer therapies [34]. In this context, several studies have focused on the antiproliferative and cytotoxic properties of natural extracts, such as phenolics, carotenoids, and tocotrienols, demonstrating their significant potential as anticancer agents [35–37].

**Figure 2:**
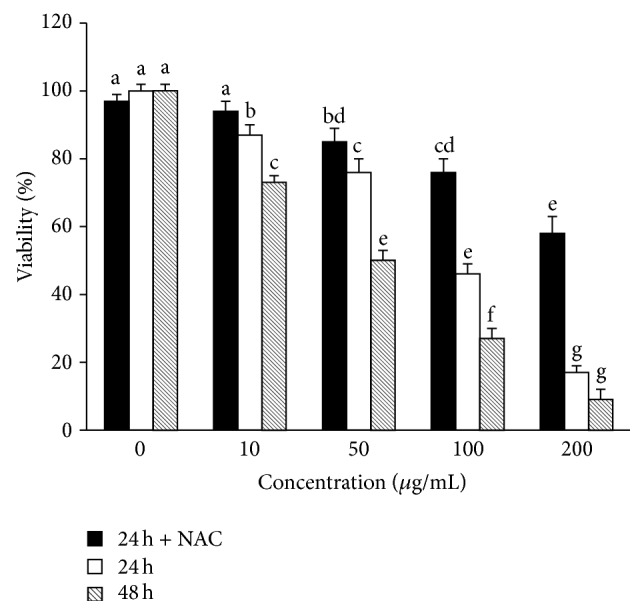
Cytotoxic effect of MRE of* S. birrea* on HepG2 cells. Cells were treated with methanolic extract at the concentration of 10, 50, 100, and 200 *μ*g/mL for 24 h, in the presence or absence of 10 mM NAC, and for 48 h. MRE inhibited the growth of HepG2 cells in a dose- and time-dependent manner. Values are means ± SD of three replicates from three independent experiments. Significant differences (*P* < 0.05) are denoted by different letters.

A previous study showed that the acetone extract of the stem bark of* S. birrea* inhibited the proliferation of different cancer cell lines (MCF-7, HT-29, HeLa) in a dose- and time-dependent manner [12]. In this study, we investigated the cytotoxicity of MRE of* S. birrea* on the hepatocarcinoma cell line HepG2 using Calcein-AM viability assay. As shown in Figure 2, the extract induced cell death in a dose- and time-dependent manner as compared with vehicle controls. The IC_50_ values at 24 h and 48 h were 109 *μ*g/mL and 42 *μ*g/mL, respectively. Cells pretreatment with 10 mM NAC, typically used as exogenously added antioxidant to lessen the potency of prooxidant polyphenols, markedly reduces MRE cytotoxic effect (IC_50_ > 200 *μ*g/mL at 24 h).

**Figure 3:**
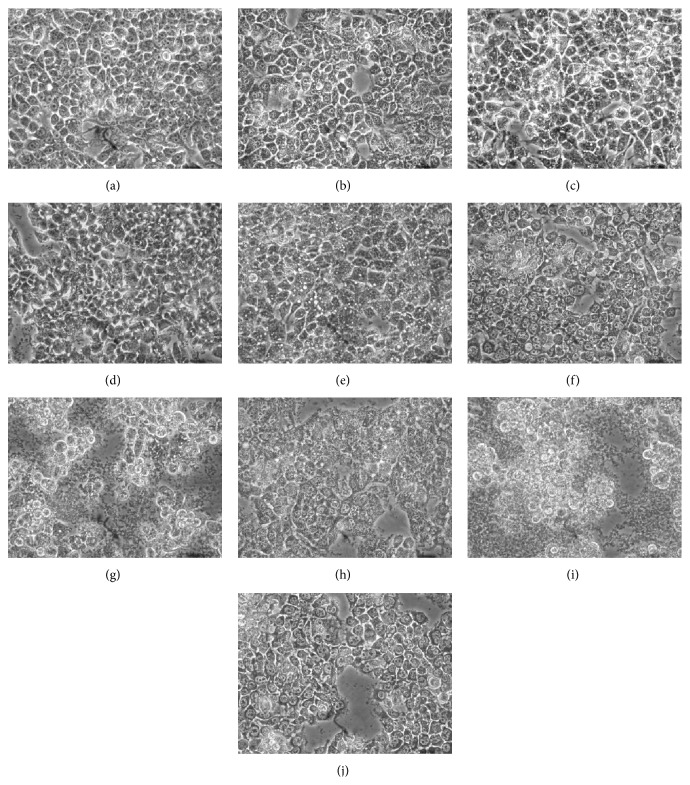
Effect of MRE exposure on HepG2 cell morphology. HepG2 cells were treated with different concentrations of root extract for 24 h and morphological changes were observed using phase-contrast microscopy. The control cells show the normal morphology. In contrast, cells treated with intermediate concentrations of MRE show abundant cytoplasmic vacuoles. At high dosage of treatment, cells became round and shrunken. The photographs were taken at a magnification ×40. Images are representative of three independent experiments ((a)-(b) control; (c)-(d) 10 *μ*g/mL; (e)-(f) 50 *μ*g/mL; (g)-(h) 100 *μ*g/mL; (i)-(j) 200 *μ*g/mL; (b), (d), (f), (h), and (j) +10 mM NAC).

Next, we evaluated the effect of MRE treatment on HepG2 cell morphology using a phase-contrast microscope. As shown in Figure 3, control cells (a) showed the normal cellular morphology, while the cells treated with different concentration of MRE for 24 h revealed remarkable morphological changes ((c), (e), (g), and (i)). Many cytoplasmic vacuoles were observed, which progressively increase in number and size, proportionally to MRE concentration. Moreover, at higher dose, the majority of cells became round-shaped and shrunken, showing blebbing or floating in the medium. Finally, cells pretreatment with 10 mM NAC considerably reduces MRE effects on HepG2 morphology ((d), (f), (h), and (j)). So, apparently, for these cells, MRE induces apoptotic-like morphology via oxidative stress.

### 3.4. Apoptosis Evaluation on HepG2 Cells

**Figure 4:**
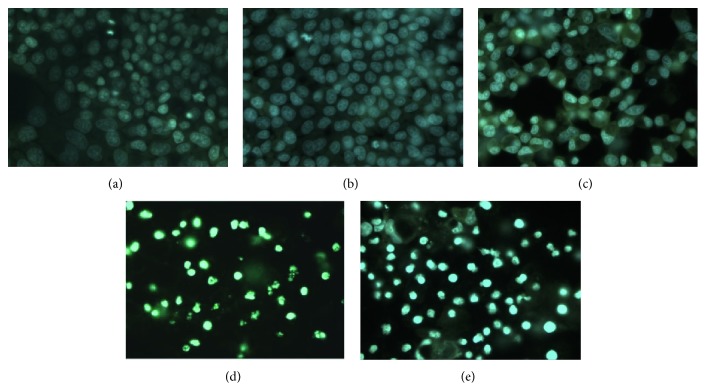
Effect of MRE treatment on the morphology of HepG2 cell nuclei. Cells were treated with vehicle (a) and methanolic root extract at 10 (b), 50 (c), 100 (d), and 200 *μ*g/mL for 24 h; cells were then stained with Hoechst 33258 and observed under a fluorescent microscope. Marked morphological changes (chromatin condensation and nuclear fragmentation) of cell apoptosis were clearly found.

To better characterize the cytotoxic effect of* S. birrea* MRE, some assays were performed on HepG2 cells treated with different MRE concentrations (10, 50, 100, and 200 *μ*g/mL) for 24 h. Firstly, the nuclear morphology was examined by staining with Hoechst 33258. As shown in Figure 4, nuclei were regular, round-shaped, and homogeneously stained in control cells (a), while the accumulation of fluorescent dye, due to morphological changes of cell apoptosis such as chromatin condensation (pycnosis), nuclear fragmentation (karyorrhexis), and cell shrinkage, was detected in treated cells in dose-dependent manner ((b)–(e)). These findings suggest that MRE treatment kills HepG2 cells via apoptotic mechanism.

**Figure 5:**
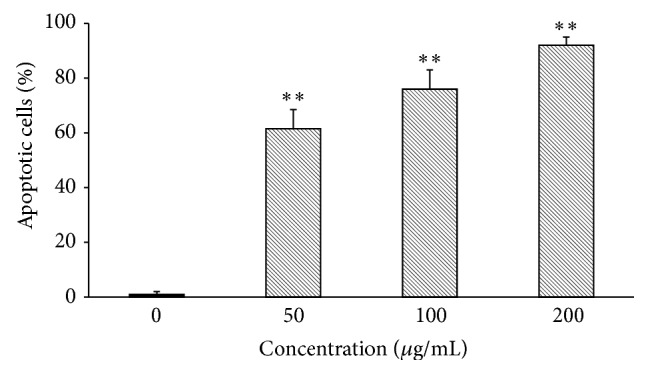
Flow cytometric analysis of apoptosis in MRE-treated HepG2 cells. HepG2 cells were incubated for 24 h with 50, 100, and 200 *μ*g/mL of methanolic root extract and apoptosis was assessed by Annexin V/7-AAD double staining. Values are means ± SD of three replicates from three independent experiments. Significant differences between the control versus treated cells are indicated by ^∗∗^(*P* < 0.01).

Secondly, the onset of apoptosis was investigated by phosphoserine biomarker staining at the cell surface. HepG2 cells were incubated with different concentrations (50, 100, and 200 *μ*g/mL) of MRE for 24 h and then stained with Annexin V-FITC/7-AAD to assess the apoptotic and necrotic cell populations. Our data show that the exposure to MRE increased the number of Annexin V-FITC-positive cells (Figure 5). In control cells, apoptotic population was 1.0 ± 0.1%; after treatment with root extract, the apoptotic rate was raised to 61.2 ± 7.1%, 76.6 ± 7.3%, and 92.0 ± 3.2%, respectively, in a dose-dependent manner.

### 3.5. Methanolic Root Extract Induces ROS Production and Reduces Mitochondria Membrane Potential (ΔΨ_*m*_)

Mitochondrial damage is a significant and early event in cellular death [38]. Elevated intracellular ROS are sufficient to trigger cell death and it has been suggested that ROS are biochemical mediators of apoptosis, mainly via interactions with proteins of mitochondrial permeability transition complex (PTPC) [39–41]. To investigate the effect of MRE on the intracellular redox status, levels of ROS production were determined after 3 h of treatment by measuring the oxidation of nonfluorescent probe DCFH-DA to its fluorescent reduced form 2′,7′-dichlorofluorescein (DCF), in the presence or not of a ROS quencher (10 mM NAC).

**Figure 6:**
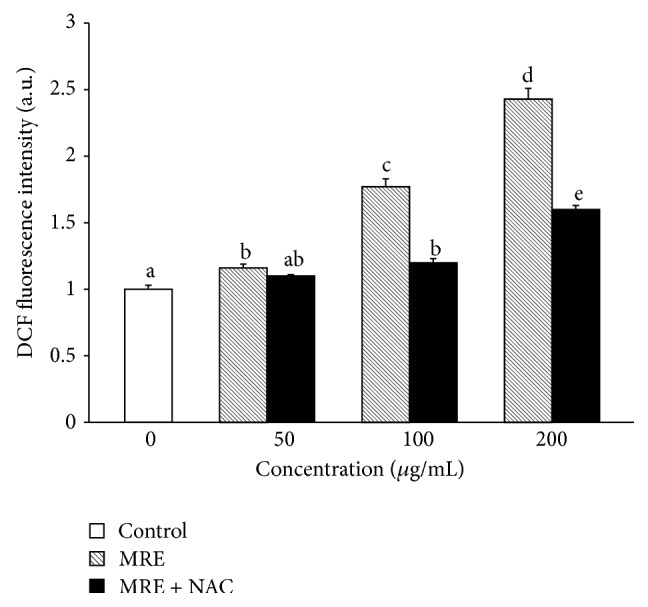
Effect of MRE on the ROS generation in HepG2 cells. Cells were incubated for 3 h with 50, 100, and 200 *μ*g/mL of methanolic root extract, generating ROS in a dose-dependent manner. NAC suppressed MRE-induced ROS generation. Values are means ± SD of three replicates from three independent experiments. Significant differences (*P* < 0.05) are denoted by different letters.

As shown in Figure 6, MRE stimulated ROS formation in a concentration-dependent manner, as compared with control cells. Moreover, pretreatment with NAC markedly inhibited apoptosis, suggesting that MRE-induced cell death is strictly related to ROS production. These data are in agreement with the increased cell viability in the presence of NAC (Figure 2). So, we speculated that high levels of ROS lead to a severe cellular damage, which directly involves the mitochondria and leads to cell death by apoptosis [41], driving these already stressed cells beyond their limit [5].

**Figure 7:**
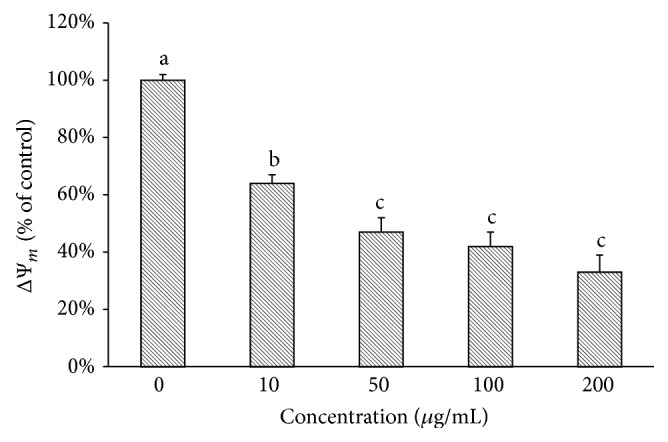
MRE-induced ΔΨ_*m*_ depolarization in HepG2 cells. The integrity of mitochondrial membranes of the cells was investigated, after 3 h of treatment, measuring TMRM fluorescence intensity of methanolic root extract-treated cells. Change in ΔΨ_*m*_ was determined by flow cytometry. Values are means ± SD of three replicates from three independent experiments. Significant differences (*P* < 0.05) are denoted by different letters.

In order to verify whether the production of MRE-induced ROS in HepG2 cells could fit with changes or loss in mitochondrial transmembrane potential (ΔΨ_*m*_), the mitochondrial membrane polarization was investigated using a cationic fluorescent probe TMRM, easily incorporated into mitochondria of viable cells. As shown in Figure 7, cells exposed to different concentrations of the methanolic root extract for 3 h showed a consistent depolarization of mitochondrial membrane potential, with TMRM fluorescence decreasing from 100% of control to 64.4 ± 1.5%, 47.7 ± 5.2%, 42.4 ± 5.3%, and 37.1 ± 6.0% of HepG2 cells treated with 10, 50, 100, and 200 *μ*g/mL, respectively.

**Figure 8:**
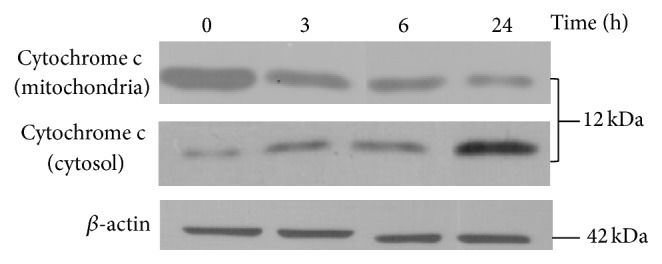
Effect of MRE treatment on cytochrome *c* in HepG2 cells. Cells were incubated for 3, 6, and 24 h with 109 *μ*g/mL (IC_50_ value at 24 h) of methanolic root extract of* S. birrea.* The cell lysates were resolved by 17% SDS-PAGE and cyt *c* expression, in both mitocondrial and cytosolic fractions, was analysed by immunoblotting.

The loss in mitochondrial membrane potential (Δ*ψ*_*m*_), an early event in apoptosis [42], represents a mitochondrial dysfunction which is one of several hallmarks of mitochondrial membrane permeabilization (MMP), together with the release of several soluble proteins (usually retained within mitochondria) in the intermembrane space (IMS), such as cytochrome *c*, Smac/DIABLO or Omi/HtrA2, with subsequent activation of effector caspases, and/or as AIF and Endo G, which are caspase-independent apoptogenic death effectors. MMP is a feature of cell death and is often considered as the “point of no return” in the cascade of events leading to apoptosis [43]. So, we investigated the release of cyt *c* from IMS to the cytosol, a key step in the mitochondrial pathway of apoptosis [44]. As shown in Figure 8, western blotting analysis reveals that MRE treatment at IC_50_ concentration caused the cytochrome *c* release into the cytosol, which increases over time (up to 24 h), in comparison to the untreated cells. Taken together, all these data demonstrate that MRE-induced apoptosis may be tightly related to loss of mitochondrial function.

### 3.6. MRE Treatment on Human Dermal Fibroblasts

**Figure 9:**
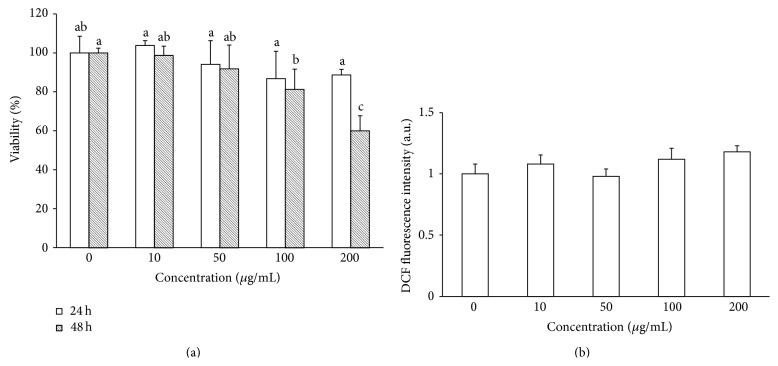
MRE treatment on human dermal fibroblasts. (a) Cells were treated with methanolic extract at the concentration of 10, 50, 100, and 200 *μ*g/mL for 24 h and 48 h. MRE shows little cytotoxicity towards fibroblasts cells. Values are means ± SD of three replicates from three independent experiments. Significant differences (*P* < 0.05) are denoted by different letters. (b) Cells were incubated for 3 h with 50, 100, and 200 *μ*g/mL of MRE, generating very low amount of ROS. Values are means ± SD of three replicates from three independent experiments. No significant differences were found (*P* > 0.05 in all cases).

Normal dermal fibroblasts provide an ideal nonneoplastic cell system to study toxicology or basic cell biology, routinely used in* in vitro* assessments [45, 46]. So, cytotoxicity and intracellular ROS levels were determined on this normal cell line treated with MRE. As shown in Figure 9(a), fibroblasts were not much affected by MRE treatment (IC_50_ > 200 *μ*g/mL in all cases), probably due to the low ROS production measured (Figure 9(b)). These data suggest that MRE treatment may be selective towards cancers cells with minimal adverse effects on normal cells.

## 4. Conclusions

In this study, our findings indicated that* S. birrea* methanolic root extract exhibits higher levels of phenolics compared to the less polar extracts, showing an important* in vitro* antioxidant activity, with particular regard to its free radical scavenging activity. Moreover, the cytotoxic effect of MRE linked to increased amounts of ROS on HepG2 cells was evident. Even if this behavior could seem contradictory, several recent papers underlined that phenolic compounds could exert both antioxidant and prooxidant activities [22, 23, 47]. In our case it is reasonable that MRE prooxidant effect could exceed its antioxidant potential on HepG2 cells. Both the loss of membrane potential and the release of cytochrome* c*, which ultimately contribute to typical morphological manifestations of apoptosis, (e.g., chromatin condensation and nuclear fragmentation) suggest that cytotoxic effect has triggered ROS-induced apoptosis in HepG2 cells, more than in human normal cells. This evidence could be associated with a cell signalling by which extract can contribute to the coordination of cell functions. The presence of different classes of secondary metabolites detected in the extract provides a preliminary explanation of the experimental evidences, suggesting the need to investigate the effects of MRE individual constituents.

Although the mechanism by which MRE treatment induces these effects remains undefined and will be the subject of further study, our findings demonstrate that compounds present in MRE are selectively able to interfere with cellular mechanisms, which are specific of malignant cells and are also linked to ROS production. This evidence suggests the potential use of MRE in therapeutic application for cancer treatment.

## Figures and Tables

**Table 1:** Total phenolic (TPC), flavonoid (TFC), and tannin (TTC) content in *Sclerocarya birrea* extracts.

Extract	TPC^*^	TFC^**^	TTC^***^
HRE	46.99 ± 1.25	7.99 ± 0.87	166.45 ± 8.01
CRE	42.77 ± 2.44	16.67 ± 2.14	215.04 ± 12.25
CMRE	100.94 ± 5.74	24.33 ± 4.78	376.30 ± 18.51
MRE	861.94 ± 12.25	95.47 ± 8.27	1109.68 ± 21.59

Root extracts: HRE (*n*-hexane); CRE (CHCl_3_); CMRE [CHCl_3_ : MeOH (9 : 1)]; MRE (MeOH).

^*^TPC was expressed as mg gallic acid equivalent/g of dried extract.

^**^TFC was expressed as mg of quercetin equivalent/g of dried extract.

^***^TTC was expressed as mg of tannic acid equivalent/g of dried extract.

Results were expressed as mean (*n* = 3) ± standard deviation; *P* < 0.01.

## Abbreviations

ABTS: 2,2′-Azinobis(3-ethylbenzothiazoline-6-sulfonic acid)
BCB: *β*-Carotene bleaching assay
BHT: Butylated hydroxytoluene
BSA: Bovine serum albumin
CMRE: Chloroform : methanol root extract
CRE: Chloroform root extract
Calcein-AM: Calcein acetoxymethyl ester
DCF: Dichlorofluorescein
DCFH-DA: 2′,7′-Dichorodihydrofluorescein diacetate
ΔΨ_*m*_: Mitochondrial membrane potential
DMEM: Dulbecco's Modified Eagle Medium
DMSO: Dimethyl sulfoxide
EDTA: Ethylenediaminetetraacetic acid
FBS: Fetal bovine serum
HRE: *n*-hexane root extract
MMP: Mitochondrial membrane permeabilization
MRE: Methanolic root extract
NAC: N-Acetyl-L-cysteine
NADH: Nicotinamide adenine dinucleotide
NBT: Nitroblue tetrazolium
PBS: Phosphate buffered saline
PMS: Phenazine methosulphate
RIPA buffer: Radioimmunoprecipitation buffer
RNS: Reactive nitrogen species
ROS: Reactive oxygen species
SDS-PAGE: Sodium dodecyl sulphate-polyacrylamide gel electrophoresis
SNP: Sodium nitroprusside
TBST: Trisbuffered saline-Tween 20
TFC: Total flavonoid content
TMRM: Tetramethylrhodamine methyl ester
TPC: Total phenolic content
TTC: Total tannin content.
